# 0915. Effect of propofol combined with opioids on guinea pig's small bowel motility in vitro

**DOI:** 10.1186/2197-425X-2-S1-O25

**Published:** 2014-09-26

**Authors:** M Schörghuber, E Tatzl, P Holzer, W Toller, S Fruhwald

**Affiliations:** Medical University of Graz, Department of Anaesthesiology and Intensive Care Medicine, Graz, Austria; Medical University of Graz, Institute of Experimental and Clinical Pharmacology, Graz, Austria

## Introduction

Critically ill patients frequently develop gastrointestinal (GI) motility disorders resulting in feeding intolerance with increased morbidity and mortality. Sedatives and opioids per se have adverse effects on GI motility and can aggravate GI motility disorders.

## Objectives

The aim of this study was to evaluate the inhibitory potency of propofol alone and in combination with remifentanil or sufentanil.

## Methods

Guinea pig´s small bowel segments of 8 cm length were set up in organ baths containing oxygenated Tyrode´s solution. Peristalsis was elicited by luminal perfusion (0.5 ml/min) against an aboral resistance of 400 Pascal (Pa). Perfusion of the segments resulted in an increase of the intraluminal pressure up to a pressure threshold (PT; mean ±SEM), where peristaltic contractions were triggered. The pressure was recorded at the aboral end of the segments. An increase of the PT was interpreted as an inhibition of peristalsis, while a decrease of the PT was interpreted as a stimulation of peristalsis. A PT of 400 Pa was equated with a complete block of peristalsis. In a first setting increasing concentrations of propofol (1; 3; 10; 30; 100; 300 µM) were added to the organ bath and the PT was evaluated at each concentration. These results were compared to increasing concentrations of propofol after pretreatment with sufentanil (0.1 resp.0.3 nM) or remifentanil (3 resp. 10 nM). Statistic calculations were performed using the general linearlized model for repeated measures and the two sided t-test of IBM SPSS 21.0.

## Results

**Figure 1:**
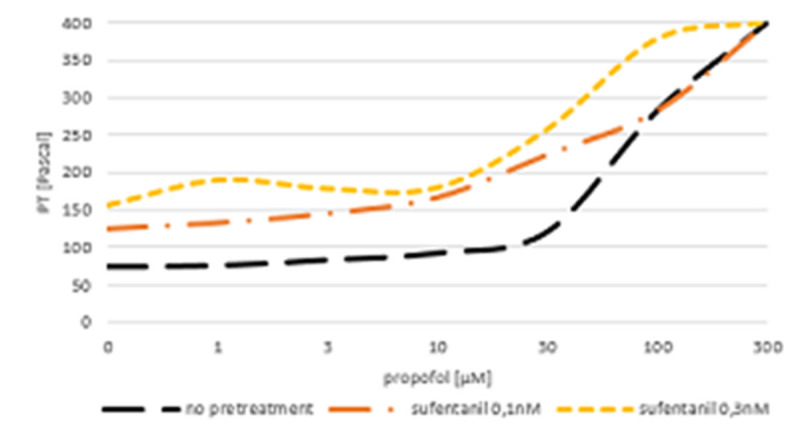
Dose-effect curve of propofol solitary and after pretreatment with sufentanil on guineapig's small bowel motility.

**Figure 2:**
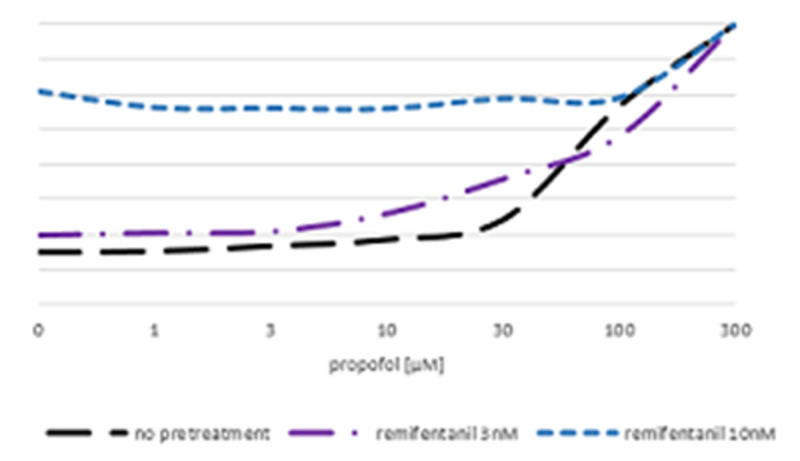
Dose-effect curve of propofol solitary and after pretreatment with remifentanil on guineapig's small bowel motility.

Basic PT without any substances added to the organ bath was 73.83 ±5.3Pa. Propofol had a dose dependent inhibitory effect on peristalsis (p < 0.001, partial ŋ^2^= 0.9; observed power= 1.00). Pretreatment with sufentanil 0.1 nM (124.2 ±16.1Pa; p= 0.039) and 0.3 nM (156.2 ±51.3Pa; p= 0.04) had an additional inhibitory effect at both tested concentrations (p= 0.041 resp. 0.029, figure 1). Remifentanil 3 nM (97.7 ±8.6Pa; p= 0.49) and 10 nM (304.2 ±49.1Pa; p= 0.005) resulted in an increase of the PT compared to basic PT with a slight aggravation of propofol's inhibitory effect (p=0.242) at low doses and a more pronounced effect at high doses (p=0.011, low vs. high concentration: p< 0.001, figure 2).

## Conclusions

Propofol and opioids affect neuronal activity via different points of action. While propofol acts on GABA receptors, opioids bind to µ, κ and δ receptors. In this experimental setting the combination of propofol with opioids shows a more pronounced inhibition of small bowel motility than propofol alone. Propofol combined with remifentanil at a high concentration had a pronounced inhibitory effect on peristalsis, while the difference between the two tested concentrations of sufentanil was minor.

